# Sugar-Sweetened Beverages and Metabolic Risk in Children and Adolescents with Obesity: A Narrative Review

**DOI:** 10.3390/nu15030702

**Published:** 2023-01-30

**Authors:** Valeria Calcaterra, Hellas Cena, Vittoria Carlotta Magenes, Alessandra Vincenti, Giulia Comola, Alice Beretta, Ilaria Di Napoli, Gianvincenzo Zuccotti

**Affiliations:** 1 Department of Internal Medicine and Therapeutics, University of Pavia, 27100 Pavia, Italy; 2 Pediatric Department, Buzzi Children’s Hospital, 20154 Milano, Italy; 3 Laboratory of Dietetics and Clinical Nutrition, Department of Public Health, Experimental and Forensic Medicine, University of Pavia, 27100 Pavia, Italy; 4 Clinical Nutrition and Dietetics Service, Unit of Internal Medicine and Endocrinology, ICS Maugeri IRCCS, 27100 Pavia, Italy; 5 Department of Biomedical and Clinical Science, University of Milano, 20157 Milano, Italy

**Keywords:** childhood obesity, sugar-sweetened beverages, metabolic syndrome, children, adolescents

## Abstract

Sugar-sweetened beverages (SSBs) are major contributors of free sugars to the diet. A strong relationship between SSB intake and weight gain is described. Methods: we performed a narrative review to present an overview of the role of SSBs as a pivotal contributor in the development of obesity and metabolism-related complications. Results: different factors influence SSB consumption in children, including economic variables, individual attributes and behaviors to environmental factors, parent features and parents’ behaviors. Data suggest that SSB intake has a negative effect on weight and obesity-related diseases. The leading mechanism linking SSB intake to the risk of gaining weight is decreased satiety and incomplete compensatory reduction in energy intake at meals following ingestion of liquid calories. Additionally, the effects of SSBs on gut microbiota and on eating behaviors were also reported. An association between SSB intake, weight gain and cardiometabolic risks is evident. Consumption of SSBs had a significant impact on the prevalence of obesity and related metabolic risks, including insulin resistance, type 2 diabetes, hypertension and metabolic syndrome. Conclusions: Limiting consumption of SSBs and increasing knowledge of the effect of SSBs on early metabolic and cardiovascular disorders will be useful in developing strategies to counteract the problem and to prevent obesity and related complications.Key future research areas for which further studies are needed include investigating the long-term effects of SSBs on health outcomes as well as analyzing the health effects of sugar consumed in solid compared to liquid forms and further elucidating the biological mechanisms of sugar addiction and energy compensation.

## 1. Introduction

The prevalence of pediatric obesity is rapidly increasing worldwide. According to a World Health Organization (WHO) report, in 2016 more than 340 million children and adolescents were in a condition of excess body weight. The Non-Communicable Disease (NCD) Risk Factor Collaboration (NCD-RisC) performed a pooled analysis of 2416 population-based studies and showed that obesity had increased by 4.9% in females (from 0.7% in 1975 to 5.6% in 2016) and 6.9% in males (from 0.9% to 7.8%) [1]. Moreover, the WHO estimated that in Europe 25–70% and 5–30% of the population presents as overweight or obese, respectively [2]. 

Childhood obesity is strongly related to socioeconomical and ethnic factors; indeed, it is more prevalent in Hispanic and African-American than in Caucasian populations, in urban areas with respect to rural ones and in subjects with a low socioeconomic level [3,4,5,6]. Prevalence of obesity is generally higher in low-income countries; however, recently this condition has also been increasing rapidly in high-income countries, whereas it tends to remain similar in developing countries [7]. Moreover, it is well known that children further gained weight due to increased food intake during lockdowns in the COVID-19 pandemic era [8]. 

The condition of obesity mainly derives from unhealthy eating habits or sedentary lifestyle and typically results from both. A diet rich in highly processed foods, with frequent consumption of SSBs, ready-to-eat snacks and fast-food preparations is an important contributing factor to the development of childhood obesity [9]. In addition, there is also a strong genetic susceptibility for this condition, which plays a permissive role and interacts with environmental factors promoting obesity [10]. Additional risk factors for obesity include sedentism, psychosocial and emotional elements, poor quality and duration of sleep and composition of intestinal bacterial flora [11]. Other conditions associated with a higher risk of excessive adipose deposits are intrauterine exposure to the mother’s excess adiposity, gestational diabetes and small-for-gestational-age (SGA) newborns [6]. During pregnancy, the differentiation of fetus hypothalamic hunger and satiety centers and adipogenesis occurs. Thus, an overstimulation of these centers during intrauterine life predisposes newborns toward overweight and obesity later in life [12].

**Figure 1:**
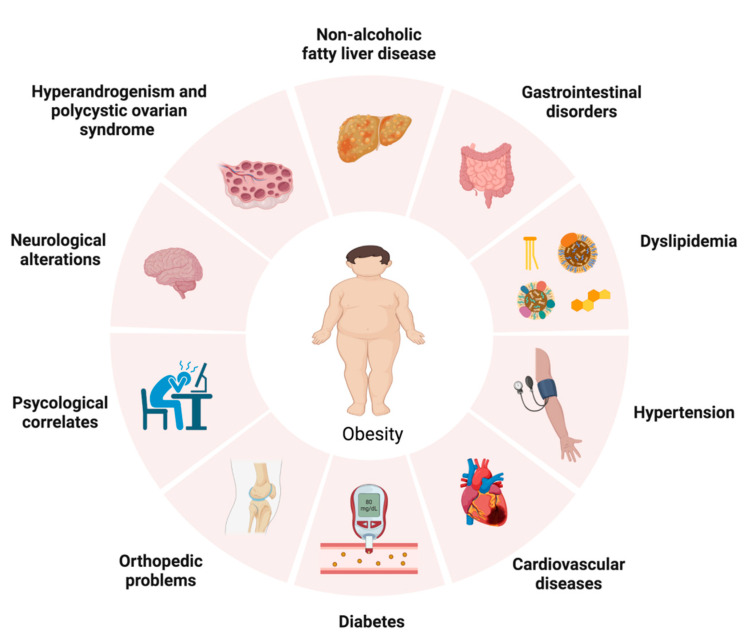
Complications related to obesity (created with biorender.com).

Pediatric obesity is a multisystem condition associated with various complications, including hyperinsulinemia and insulin resistance (IR), type 2 diabetes mellitus (T2DM), hypertension, dyslipidemia, hyperandrogenism and polycystic ovarian syndrome (PCOS), chronic inflammation, endothelial dysfunction, asthma and obstructive sleep apnea syndrome (OSAS) [13,14,15,16,17,18], gastrointestinal [19] and neurological disorders (such as idiopathic endocranial hypertension, migraine and chronic headache [20,21,22,23,24]) and psychosocial complications [25,26,27]. The complications related to obesity are depicted in Figure 1.

Metabolic complications are more relevant health risks for children with obesity [1]. Among the factors shown to contribute to the increase in obesity and these deleterious consequences, sugar-sweetened beverages (SSBs) have recently received a great deal of attention [28,29,30,31,32]. 

Consumption of SSBs has dramatically increased among children and adolescents in the past decades, becoming a regular drink of choice for millions around the world [32]. Compounding the problem is that SSB portion sizes have risen dramatically in recent years, leading to additional increased consumption among youths [33]. According to the WHO, the term “sugar-sweetened beverages” defines all beverages containing free sugars [34]. National authorities have also provided different definitions considering the sugar content per volume, which have been utilized for regulatory purposes such as taxation [35].

SSBs are major dietary source contributors of free sugars [36]. A typical 12-fluid-ounce serving of soda contains 35.0–37.5 g of sugar and 140–150 calories [35]. 

Even though a definite causality has not been detected, a strong relationship between SSB intake and weight gain has been described. Furthermore, literature evidence suggests that increased SSB intake is related to T2DM, metabolic syndrome and cardiovascular risk starting in childhood and adolescence.

We present an overview of consumption of SSBs and their role as contributors to the development of obesity and related pediatric metabolic risk. We describe the global trend in the intake of SSBs, the factors that influence their consumption and the mechanism by which SBBs impact childhood heath, focusing on the relationship between SSBs, calorie intake and weight gain and related complications in order to raise awareness of the negative impact of SSBs among clinicians and different professionals. Indeed, an increase in knowledge of the effect of SSBs on early metabolic and cardiovascular disorders, starting in childhood, will be useful in developing strategies to counteract the problem and to prevent obesity and related complications. 

## 2. Methods 

**Figure 2:**
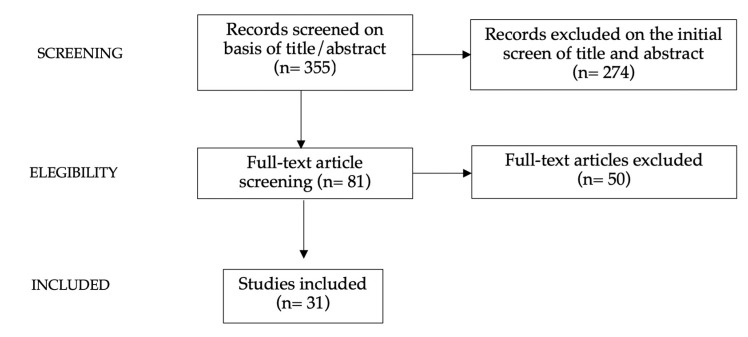
Studies selected.

A narrative review was proposed [37] in order to offer a condensed and filtered overview on the intake of SSBs and the effects of SSB consumption on the development of obesity and correlated metabolic complications. Authors V.C.M., G.C., A.V. and A.B. independently reviewed relevant English-language literature from the last 15 years. Original papers, meta-analyses, clinical trials and reviews were included in the research (Filter: 0–20 years). Case reports, case series, brief reports, commentaries and editorials were excluded. The PubMed, Scopus, and Web of Science databases were adopted for the research. The following keywords (alone and/or in combination) were used for the research: obesity, children, sugar-sweetened beverages, nutrition, diet, metabolic syndrome, health consequences and obesity-related diseases. Authors V.C.M., G.C., A.V. and A.B. assessed the abstracts of the available literature (n = 355), screened the full texts of potentially relevant articles (n = 81) and reviewed, analyzed and discussed relevant full texts (n = 31). The characteristics of the selected studies are reported in Figure 2. The reference lists of all manuscripts were also checked to identify relevant studies. The resulting draft was critically discussed by C.V., H.C. and G.Z. All authors approved the final version.

## 3. Results

In Table 1, we summarize the manuscripts analyzed to discuss the specific topic of this review. 

## 4. Discussion of the Results

### 4.1. Sugar-Sweetened Beverage Consumption and Mechanism to Impact Health Outcomes

#### 4.1.1. SSB Consumption and Associated Factors

SSBs are consumed widely on a global scale. While data for SSB consumption exist for individual countries, few reports on SSB consumption collate and compare such data globally among children and youth. 

According to a 2021 report [69] from the European Union’s statistical office, in European Union (EU) countries, 14% of adolescents and young adults (15–24 years old) consume SSBs at least once a day. In the US, according to the “What We Eat in America” analysis (NHANES 2013–2016) [70], 24% of added sugar intake came from SSBs in people older than 1 year old, specifically 16% from soft drinks, 5% from fruit drinks, 2% from sport and energy drinks and 1% from other sources [71]. A more comprehensive systematic review (64 included countries) examining adolescent consumption of vegetables, fruit, carbonated soft drinks, and fast food reported that adolescents (aged 12 to 17 years) consumed SSBs approximately 0.99 times daily [72]. 

Although SSB consumption still remains high in many high-income countries, a decreasing trend considering specific age ranges can be noticed, while studies show increased levels of SSB consumption in low- and middle-income countries [35].

Over the last two decades, in high-income countries (i.e., Canada, Australia, South Korea, UK), consumption has decreased or remained stable in children (age range 1–11) and increased in adolescents (age range 12–19) [73]. On the other hand, in middle-income countries such as Mexico, a decrease in intake was found only in the youngest (1–5 years old), while in children (6–11 years) and adolescents (11–19 years old) a significant increase was detected [73]. These upward trends can be explained by urbanization, economic growth and economic transition in poor countries, which has facilitated access to and consumption of SSBs [35,73].

The framework of factors that influence SSB consumption in children includes a large number of variables in addition to economic ones, which range from individual attributes and behaviors to environmental factors (e.g., available food supply, food-related government policies, food advertising) and parent features (e.g., food-related knowledge, socioeconomic status, weight status) and parents’ behaviors (e.g., food preparation and consumption modeling) [74]. Indeed, SSB consumption in children was strongly associated with parents’ consumption in European and Australian studies [75]. Pettigrew et al. investigated parents’ (n = 1302) and their children’s (8–14 years old) eating behaviors and found that children’s soft drink consumption was mainly associated with parental attitudes toward SSBs, compared with other variables (i.e., amount of time spent watching television, the extent of pestering they experience from their children and their perceptions of others’ approval of the consumption of SSBs) [74].

Since parents are role models for their children, the way the former eat shapes the eating behavior of the latter [43,76]. Other aspects also need to be considered, such as that parents are responsible for the availability of food at home and therefore their choices are critical in determining healthy eating behavior in children. This is evidenced by a cross-sectional study designed to determine whether there is a relationship between parents’ and children’s eating patterns (n = 1662 mothers and n = 789 fathers; children 6–16 years) [77]. Low parental educational level and low socioeconomic status are all strongly associated with children’s SSB consumption, as they are all factors influencing food availability at home [76,78,79]. In support of this, a Canadian study conducted to identify environmental and sociodemographic correlates of SSBs in preschool children (n = 2114, aged 4 and 5 years) found that children living in low-SES (socioeconomic status) neighborhoods were significantly more likely to have consumed SSBs within the last week [80].

#### 4.1.2. SSBs and Metabolic Impact 

The relationship between SSB consumption and risk for gaining weight, obesity and related comorbidities is well-documented in the literature [15], but how it occurs is still not entirely clear.

The leading mechanism linking SSB intake to risk of gaining weight is decreased satiety and incomplete compensatory reduction in energy intake at meals following ingestion of liquid calories [81]. Short-term trials showing greater energy intake [38,39,82] and weight gain [38,81] upon SSB intake illustrate this point.

Vien et al. [38] conducted two experiments in children (age range 9–14) with normal weight, overweight or affected by obesity, using a randomized repeated-measures design. The aims were to (i) evaluate the effects of isocaloric (130 kcal) amounts of 2% milk, 1% chocolate milk, 1.5% yogurt drink, an SSB (fruit punch) and water consumed before and during a pizza meal 60 min later on satiety and food intake (experiment 1) and (ii) to compare the effects of 2% milk and an SSB consumed before and during a pizza meal 60 min later on food intake, glycemia and appetite hormone responses (experiment 2). In experiment 1, meal food intake was lower after the yogurt drink and chocolate milk (but not milk) compared to water [38]. After caloric beverages, energy intake was higher than water consumption [38]. In experiment 2, post-meal glucose was 83% higher in children with overweight/obesity than normal weight subjects [38]. Milk led to higher pre-meal GLP-1 and post-meal PYY than the SSB, without difference in insulin level [38]. Compared to SSBs, dairy products consumed before and with a meal had more effect on appetite, food intake and satiety hormones, but all caloric beverages resulted in more total calories than if water was the beverage. 

Maersk et al. [39], while examining the effect of two different isocaloric but macronutrient beverages (SSBs versus semi-skimmed milk) and two non-energy-containing beverages (aspartame-sweetened diet soft drink and water) on energy intake, appetite and appetite-regulating hormones in 24 subjects affected by obesity, found that energy contained in beverages is not compensated by decreased energy intake at the following meal, and so total energy intake is higher when SSBs are consumed. Semi-skimmed milk induced greater subjective fullness and less hunger than SSBs. Milk led to 31% higher GLP-1 and 45% higher GIP concentrations compared with SSBs [39]. Ghrelin was equally 20% lower after milk and SSBs compared with water [39]. The total energy (ad libitum energy intake plus energy intake from the drink) was significantly higher after the energy-containing drinks compared with the diet soft drink and water [39].

SSBs contribute to the progress of chronic disorders such as T2DM and cardiovascular diseases partially through their ability to favor weight gain but also indirectly through the effects of constituent sugars. 

SSB consumption results in an increase both in blood glucose and insulin levels [83]. In general, SSBs have moderate-to-high glycemic index values [84] and the large quantities consumed contribute to a high dietary glycemic load, inducing glucose intolerance and IR [85]. In addition, because of their high quantities of rapidly absorbable carbohydrates such as HFCS (the primary sweetener used in SSBs) and the high quantities consumed, SSBs may be associated with higher risk of T2DM [80] and impairment of β-cell functions [86]. 

Several studies also suggest that consuming fructose, present both in sucrose and HFCS in similar amounts, may have particularly adverse effects on selective deposition of visceral fat, lipid metabolism, de novo lipogenesis and insulin sensitivity [35]. 

Fructose is metabolized in the liver and converted to glucose, lactate and fatty acids when consumed in moderate amounts [87]. Excessive fructose consumption can lead to increased hepatic de novo lipogenesis, dyslipidemia and IR [88,89].

The hepatic metabolism of fructose can also deplete intracellular adenosine triphosphate in hepatocytes, leading to an increase in uric acid production [35], since fructose is known to increase the production of hepatic uric acid. Hyperuricemia is a precursor to gout [90,91] and both gout and hyperuricemia have been associated with hypertension, MetS, T2DM and CVD later in life [92,93].

#### 4.1.3. SSBs and Impact on Gut Microbiota 

As already mentioned, the excess sugar intake from SSBs has been hypothesized to be causally linked to numerous modern diseases [35]. The link between these chronic diseases and sugar has been hypothesized to be at least partially via the gut microbiome (GM) [94].

The GM is a crucial actor that has close interplay with the host metabolism and produces various bioactive metabolites, such as short-chain fatty acids, phenols, ammonia, amines and sulfides [95]. Microbiota-derived metabolites act as nutrients and messenger molecules and can signal organs in the body to shape host pathophysiology [96,97]. Dietary factors are a determinant of GM diversity and can modulate gut bacterial communities, as shown from the microbial modulation observed in response to probiotic and prebiotic treatment as well as the dysbiosis resulting from consuming unhealthy yet palatable foods such as SSBs that are associated with chronic diseases [98,99,100].

In a review, Di Rienzi et al. [94] summarized different potential pathways for how our GM may adapt to the high intake of sugars. Compositional, transcriptional and genetic changes can cause modifications within the bacterial strains, all with the purpose of adjusting to better utilize the present substrate. Despite this, few studies reported that the intake of SSBs could influence GM composition, with a lack of studies focusing on children and youth. 

Ramne et al. [40] reported that *Lachnobacterium* was significantly inversely associated with SSB intake, even though this genus has rarely been studied and has never been associated with any cardiometabolic traits [40]. A positive association between SSB intake and the Firmicutes/Bacteroidetes ratio after adjusting for both BMI and fiber intake was also observed [40].

Yan et al. [41] assessed cross-sectional associations between self-reported SSB intake, circulating microbial metabolites and GM-host co-metabolites and metabolic health outcomes; obesity-related markers and blood lipids were positively associated with SSB consumption [41]. Moreover, 79 microbial metabolites and GM host co-metabolites were identified that were associated with both SSB intake and metabolic health outcomes, independent of potential cofounding factors, and were enriched in pathways of branched chain amino acid catabolism and aminoacyl-tRNA biosynthesis [41]. SSB intake may affect obesity and cardiometabolic straits via regulating amino acid biosynthesis in relation to GM as reported by other studies [95,100].

Collectively, studies have proven associations between habitual SSB intake and metabolic markers linked to chronic and degenerative diseases, highlighting the pivotal involvement of GM-related metabolites in mediating such associations [41].

#### 4.1.4. SSBs and Dietary Behavior and Emotional Eating

Dietary behaviors are a complex concept, developed from psychological and social perspectives, including food choices and preferences, hedonic response, food acceptance and neophobia (a reluctance to try novel foods) [42]. 

Although SSBs have been considered one of the dietary factors with the greatest impact on childhood health overall, the effects of SSB consumption on eating behaviors are poorly studied. 

Some cross-sectional studies have evidenced that SSB consumption is positively associated with food approach behaviors and negatively associated with food avoidant behaviors [42]. 

Elfhag et al. [43], while investigating associations between consumption of sweets, SSBs, fruits and vegetables and the psychological dimensions of eating in Swedish 12-year-old children, found a lower SSB consumption among children with higher response to internal satiety cues. 

In contrast, studies in Finland [44] and the Netherlands [45] showed no relationship between SSB consumption and appetitive traits of food approach among school-age children. 

In the Finnish PANIC study [44], the authors investigated whether eating frequency and food consumption (assessed by a 4-day food record) are influenced by eating behavior (assessed by the Children’s Eating Behavior Questionnaire) in a sample of 406 school children. Results reported no association between a higher score in the Desire to Drink subscale and higher consumption of SSBs [44]. The same results were reached by Rodenburg et al. [45] using data from 1275 children participating in the IMPACT study in 2009–2010 [45].

Recently, the role of sugar addiction as a driver of excessive SSB intake has been considered. Sugar consumption has been shown to release opioids in the nucleus accumbens, a crucial site for reinforced behaviors in the brain, and to stimulate the reward system [101,102]. 

Despite evidence from studies in animal models, a variety of reviews on this topic are conflicting when considering results in humans [103].

### 4.2. Sugar-Sweetened Beverages, Weight Gain and Metabolic Complications 

There is indeed mounting evidence that higher obesity levels are related to the increased SSB intake among children and adolescents over time [30,73,104]. 

Indeed, US children consume nearly twice as many calories from SSBs compared to 30 years ago [28,29], and SSBs are the largest category of caloric intake in the pediatric population [28,30]. SSB consumption differs by age range and begins at a young age [28,73,104,105,106,107]. Consumption patterns in preschool children are of particular concern. Bleich et al. [105], analyzing NHANES data from 2007 to 2010, reported that daily SSB consumption was present in 62% of preschool children aged 2–5 years, 73% of children aged 6–11 years and 76% of adolescents aged 12–19 years [105]. Almost half (44%) of toddlers 1.5 to 2 years old were reported to consume an SSB on any given day [28,104].

Parallel to SSB intake, a gradual rise in SSB calories consumed was also evidenced among SSB drinkers. Indeed, Bleich et al. showed that children aged 2–5 years consumed 127–139 kcal from SSBs per day, children aged 6–11 years 176–220 kcal per day and adolescents 290–298 kcal per day [105]. SSB consumption varies not only according to age group but also by racial/ethnic group, socioeconomic status (SES) and sex [107,108,109]. Interestingly, at younger ages (2–5 years), African American children consumed a higher quantity of SSBs compared to Hispanic, white and Asian children, whereas among adolescents (12–19 years), white children consumed the highest quantity of SSBs, followed by Hispanics, African Americans and Asians [107,110]. Considering SES, children followed from ages 2 to 5 years in the lowest SES quintile consumed a higher quantity of SSBs on a daily basis compared with those in the highest SES quintile (16–23% versus 4%) [105,107].

#### 4.2.1. SSBs and Weight Gain

Research about SSB consumption has mostly focused on the correlation with unhealthy weight status (higher BMI and higher amount of adipose tissue). A variety of studies (cross-sectional, longitudinal and interventional) considered the relationship between SSBs and weight gain.

Cross-sectional studies cannot determine causality between SSB intake and weight gain but may provide useful information on relationships. Specifically, it has been shown that Hispanic American children (250 kindergarten students) who regularly consumed SSBs had an odds ratio (OR) of obesity of 3.7. In the group studied, overweight children were more likely to be SSB drinkers [46]. Moreover, Scharf et al. found a relationship between the number of SSB servings and the BMI z-score of the subjects at 4 and 5 years of age [109]. After adjustment for confounding variables (such as SES, sex and ethnicity), the authors showed higher odds of overweight and obesity at age 4 and 5, respectively [109]. In 4283 Australian children, Grimes et al. found that those drinking SSBs were more likely to be overweight [47]. These results were further confirmed by Nicklas et al., who evaluated children involved in the Bogalusa Heart Study at age 10 and found an OR for overweight of 1.33 for SSB consumers compared to non-SSB-drinkers [48]. More recently, Gui et al. evaluated a national sample of children and adolescents in China and investigated the association between SSBs and obesity and hypertension [106]. The authors showed that the SSB consumers among the participants had a higher OR (1.133, 95%) than non-consumers for abdominal obesity (after adjustment for age, sex, SES, diet, screen time and physical activity) [106]. In addition, Davis et al. showed that high intake of SSBs in children with obesity was linked to increases in obesity prevalence; on the contrary, no SSB intake was associated with a 28% reduction in obesity prevalence [50]. Finally, Gallagher et al. evaluated a sample of 2665 Greek school children, assessing body composition, anthropometric parameters, dietary intake and serum cortisol data and observed a positive association between high SSB consumption (>2 servings per day) and visceral adipose tissue [51]. Nevertheless, not all studies agree on this relationship. For instance, O’Connor et al. evaluated 1160 children (ages 2–5 years) from the 1999–2003 NHANES cohort and did not find higher BMI in children drinking >12 ounces daily of SSBs compared to non-drinkers [52]. Moreover, Keller et al. did not find any difference in BMI according to SSB intake evaluating a small cohort of children ages 3–7 years [53]. The ability of SSBs to increase weight mainly depends on whether the calories ingested from SSBs exceed the individual’s usual caloric intake. 

Among longitudinal studies, potentially able to identify associations between SSB consumption and changes in weight status over time, Dubois et al. showed that children who drank SSBs between the ages of 2.5–4.5 years had an OR of 2.4 for being overweight at age 4.5 years compared to non-drinkers [54]. Moreover, De Boer et al., analyzing data from children followed between the ages of 2 and 5 years, noticed that those consuming ≥1 SSB daily had a greater change in BMI z-score and were more likely to become overweight and obese over the next 2 years when compared to children drinking fewer SSBs [109]. In addition, a meta-analysis of longitudinal studies evaluated 22 studies that analyzed the risk of overweight development between children with a daily intake of SSBs versus none. The authors showed an OR of 1.55 for higher levels of BMI among SSB drinkers over time [55].

Pan et al. studied the correlation between SSB intake in infancy and obesity prevalence at age 6 [56]. The odds for obesity detected were 71% higher for any SSB intake and 92% higher for SSB introduction before age 6 months (compared with subjects without SSB intake in infancy) [56]. 

Another longitudinal, multicenter study by Zheng et al. studied associations between SSB consumption when in pediatric age range and subsequent changes in body fatness in early adulthood [57]. The authors enrolled children aged 9 years and evaluated data at three time points: 9, 15, and 21 years; subjects who consumed more than 1 serving of an SSB per day at age 15 years had higher BMI and waist circumference than non-drinkers in the subsequent 6 years. Moreover, children who increased SSB intake from age 9 to 15 years had larger increases in waist circumference and BMI from 15–21 years compared to those with no increase in SSB intake [57]. Even among longitudinal studies, not all results are coherent. For example, neither Laurson et al., who followed a cohort of children (10-year-olds) for 18 months, nor Forshee et al., who performed a meta-analysis of 12 longitudinal studies among children aged 2–19 years old, found an association between SSB consumption and weight gain [58,59]. 

To provide stronger evidence of a causal link between SSB consumption and weight gain, different interventional studies evaluated weight differences among children and adolescents randomized to consume either SSBs or non-caloric alternatives [109]. Specifically, Ebbeling et al. performed a large-scale trial, randomizing 224 adolescents either to receive no intervention or to have non-caloric beverages, with instructions to drink these beverages instead of SSBs. The researchers reported that at one year follow-up, adolescents who received non-caloric beverages gained less weight and had lower BMI compared to those continuing with their usual SSB intake [60].

De Ruyter et al. tested the effects of substituting SSBs with non-caloric beverages in a double-blinded randomized trial in children ages 4–11 [61]. Children randomized to receive non-caloric drinks (versus similar SSBs in a blinded fashion) had gained less weight by 18 months; indeed, in this group, lower BMI z-scores, smaller waist circumferences and lower fat percentages were observed [61]. 

These reports demonstrate that calorie removal from SSBs is effective at reducing an increase in weight gain and BMI, further highlighting that calories consumed from SSBs tend to exceed the individual’s usual caloric intake, promoting weight accumulation.

#### 4.2.2. SSBs and Type 2 Diabetes Mellitus

Current evidence suggests a relationship between SSB intake, obesity, and T2DM. Scientists proposed different potential mechanisms for this link, mainly in the adult population. Specifically, SSBs have been shown to contribute to weight gain mainly because of their high sugar content, low satiety and potential incomplete compensation for total energy that may lead to increased energy intake [7,108]. Moreover, these beverages have also been shown to contribute to T2DM development due to their high quantities of rapidly absorbable carbohydrates (such as the high-fructose corn syrup used in SSBs) and to their high dietary glycemic load, which leads to inflammation, IR and impaired pancreatic β-cell function [28,49,86]. The majority of research on this topic involves adults, and pediatric studies mainly focus on obesity development upon regular intake of SSBs. A large cohort study by Abbasi et al. found that a child with obesity faces a 4-fold greater risk of T2DM diagnosis by age 25 than a normal-weight child [111]. A meta-analysis by Malik et al. also found a relationship between SSB intake and T2DM [86]. The authors reported that subjects in the highest quantile of SSB intake (1–2 servings/day) had a 26% greater T2DM risk with respect to individuals in the lowest quantile (none or <1 serving/month) [86]. Unfortunately, the studies focusing on SSB intake during childhood and adolescence and later T2DM development are scarce; thus, no definitive conclusions can be drawn. However, in adults, SSB intake is associated with an increased T2DM risk and relevant adverse health effects [112,113] and two important meta-analyses evidenced a strong association between SSB intake and T2DM development [113,114,115]. 

#### 4.2.3. SSBs, Hypertension, Cardiovascular Risk and Metabolic Syndrome 

The impact of SSBs on blood pressure (BP) has been debated; evidence suggests that increased SSB intake is related to higher risk of developing hypertension (HTN). The role of sugars in developing cardiometabolic disorders and HTN, already well known in adults [62], has only recently been actively investigated in children, specifically in recent works in the pediatric field focused on the role of SSBs in developing hypertension [63,88,116,117,118]. Farhangi et al. reported that high SSB consumption was associated with a 1.67 mmHg increase in systolic BP (SBP) in children and adolescents but not with a significant rise in diastolic BP (DBP). High SSB consumers were moreover 1.36 times more likely to develop HTN compared with low SSB consumers [63]. According to their findings, high SSB intake increases SBP and HTN risk in the pediatric age range.

Different studies also evaluated a correlation between dietary intake of sugars and markers of cardiovascular disease in children and adolescents [64,65,113,119,120]. Pollock et al. showed that higher total fructose consumption in adolescents was associated with cardiovascular disease and T2DM risk [64]. Welsh et al. reported a positive association between consumption of added sugars and multiple cardiovascular risk markers [65]. Specifically, added sugar intake was positively correlated with low-density lipoprotein cholesterol (LDL) and triglyceride (TG) levels and negatively with mean high-density lipoprotein cholesterol (HDL) levels. Moreover, the authors positively correlated added sugar intake and IR index in adolescents with overweight and obesity [65].

Recently, Zhu et al. examined a possible relationship between SSB intake and cardiometabolic risks among a Chinese pediatric population [66]. The researchers evaluated 3958 subjects (aged 6–17 years) and requested a 3-day dietary record and a food-frequency questionnaire to precisely assess SSB consumption [66]. The authors measured anthropometric and laboratory parameters to evaluate cardiometabolic indicators: SSB intake was positively correlated to serum total cholesterol and LDL. Furthermore, the participants in the highest intake category of SSB consumption (≥201.7 mL/day) had 0.10 mmol/L higher total cholesterol and 0.09 mmol/L higher LDL levels than the non-consumption group [66]. 

High intake of SSBs in adults was also demonstrated to increase the risk of metabolic syndrome (MetS) [86,88]. Mirmiran et al. assessed the consumption of SSBs in relation to incidence of MetS [121,122] among children and adolescents [67]; compared to the first quartile of SSB intake, the OR of MetS in the highest quartile was 3.20 [67]. For incidence of MetS components, the highest quartile of SSDs showed an OR of 2.49 for abdominal obesity and 2.79 for HTN (compared with the lowest quartile) [67]. Li et al. recently evaluated the association of SSB intake with MetS risk among children and adolescents in urban China [68], showing that subjects with high SSB intake were at higher risk of MetS (OR = 1.60) and abdominal obesity (OR = 1.55) compared with their participants with no SSB intake [68].

Although there is evidence agreeing on the negative impact of children’s SSB intake on BP, cardiometabolic risks and MetS, further research would be interesting as well as the evaluation of useful strategies to counteract the problem.

## 5. Conclusions

SSB intake has a negative effect on weight and obesity-related diseases. In particular, an association between SSB intake and cardiometabolic risks is evident. Limiting consumption of SSBs will have a significant impact on obesity prevalence and metabolic risk in children and adolescents. 

Consistent with the recommendations of limiting intake of sugar or free sugar, a number of public policies involving multiple stakeholders and public–private partnerships is required to help change SSB consumption patterns.

Fiscal incentives to reduce SSB intake, such as taxation, should be considered by governments. Taxes should be comprehensive, covering all SSBs, and it is better for consumers to consider the price of the product with the tax included instead of adding the tax only at the time of purchase.

Tax policies should be combined with regulatory standards, including the promotion of new, clear, practical front-of-package labeling design for children and adolescents to improve youth empowerment. 

Regulatory action to reduce the marketing of unhealthy beverages such as SSBs in the media should be also be taken into consideration. 

Further actions should be the limitation of access to SSBs in settings that represent excellent contexts for healthy lifestyle interventions in children and adolescents, such as schools and sport locations. Access to SSBs should be restricted in schools by adopting policies that limit sales and provision of SSBs in school meal programs; on the other hand, access to water should be prioritized, with policies and regulatory actions that ensure access to potable water. 

Healthy hydration represents a crucial aspect of a healthy life; however, the recommendations for beverage choices are still less detailed compared to food-based recommendations. 

Future health education and the promotion of behaviors to avoid SSBs are mandatory for preserving children’s health. 

Key future research areas for which further studies are needed include examining the long-term effects of SSBs on health outcomes, investigating the effects of sugar in solid form compared to liquid form and further elucidating the biological mechanisms of sugar addiction.

Reducing SSB consumption remains a crucial step in improving quality of diet, which could have a measurable effect at an individual level and on improving global health.

## Figures and Tables

**Table 1:** Manuscripts analyzed to discuss sugar-sweetened beverage consumption and its impact on weight gain and health outcomes.

Authors	Journal/Year	Study Design	Population Involved (Sample Size and Age)	Type of SSBs Studied	Main Results
Vien et al. [38]	Appl. Physiol. Nutr. Metab. 2017	Interventional randomized controlled trial (two experiments)	32 children, 6–14 years old (experiment 1) 20 children, 6–14 years old (experiment 2)	Fruit punch	SSB consumption has negative effects on food intake, appetite and satiety hormones compared to other beverages tested (dairy products)
Maersk et al. [39]	Eur. J. Clin. Nutr. 2012	Cross-over	24 subjects, 20–50 years old	Sucrose-sweetened regular cola	Total energy intake (ad libitum energy intake plus energy intake from the drink) was higher after the energy-containing drinks compared with diet cola and water (*p* < 0.01). SSBs induced lower subjective fullness and higher hunger than other beverages tested (still water and diet cola).
Ramne et al. [40]	Eur. J. Nutr. 2021	Cross-sectional	1371 Swedish participants, 18–70 years old	Any type	Negative association between SSB intake and *Lachnobacterium.* Positive association between SSB intake and Firmicutes/Bacteroidetes ratio after adjusting for both BMI and fiber intake.
Yan et al. [41]	Nutr. Metab. Cardiovasc. Dis. 2022	Cross-sectional	86 young Chinese adults, age = 23 ± 4 for men and age = 22 ± 3 for women	Carbonated beverages, fruit juice, lactobacillus drinks, energy drinks, tea drinks, bubble tea, pre-sweetened coffee, milk, yogurt and other dairy drinks.	Carbonated beverages, fruit juice, energy drinks, and bubble tea exhibited positive associations with obesity-related markers and blood lipids.
Sweetman et al. [42]	Int. J. Behav. Nutr. Phys. Act. 2008	Cross-sectional	346 same-sex twin English preschoolers, 9–12 years old	Sweetened soft drinks, fruit juice, fruit squash	Higher scores on the Desire to Drink subscale, measured through the Children’s Eating Behaviour Questionnaire, were associated with higher preferences and greater frequency of SSB consumption.
Elfhag et al. [43]	Public Health Nutr. 2008	Cross-sectional	1441 children, approximately 12 years old	Soft drinks	Lower SSB consumption among children with higher response to internal satiety cues.
Jalkanen et al. [44]	Appetite 2017	Cross-sectional	406 children, 6–8 years old	Carbonated and non-carbonated beverages	No association between higher score in Desire to Drink subscale and higher consumption of SSBs.
Rodenburg et al. [45]	PLoS ONE 2012	Cross-sectional	1275 children from the IMPACT study in 2009–2010, 9 years in 2009	Any type	No consistent associations were found between children’s appetitive traits and SSB intake.
Ariza et al. [46]	J. Urban Health Bull. N.Y. Acad. Med. 2004	Cross-sectional	250 kindergarten students, 5–6 years old	Any type (excluding fruit juice)	Children who regularly consumed SSBs had an odds ratio (OR) of obesity of 3.7.
Grimes et al. [47]	Pediatrics 2013	Cross-sectional	4283 Australian children and adolescents, 5–6 years old	Any type	Children drinking SSBs 26% more likely to be overweight/obese (OR 1.26, 95% CI: 1.03–1.53).
Nicklas et al. [48]	Am. J. Prev. Med. 2003	Cross-sectional	562 children aged 10 years (Bogalusa Heart Study)	Soft drinks, fruit-flavored drinks, tea and coffee	OR for overweight of 1.33 for SSB consumers compared to non-SSB-drinkers.
Gui et al. [49]	Nutrients 2017	Cross-sectional	53,151 children and adolescents aged 6–17 years	Coca-Cola, Sprite, orange juice, Nutrition Express, and Red Bull	SSB consumers among the participants had a higher OR (1.133, 95%) than non-consumers for abdominal obesity.
Davis et al. [50]	Obesity 2014	Cross-sectional	2295 low-income children 2–4 years old	Soda and fruit drinks	High intake of SSBs was linked to increases in obesity prevalence; on the other hand, no SSB intake was associated with a 28% reduction in prevalence of obesity.
Gallagher et al. [51]	Br. J. Nutr. 2021	Cross-sectional	2665 Greek school children aged 9–13 years	Any type	Positive association between high SSB consumption and visceral adipose tissue (*p* = 0,01).
O’Connor et al. [52]	Pediatrics 2006	Cross-sectional	1160 children aged 2–5 years from the 1999–2003 NHANES cohort	Any type (including 100% fruit juice)	No higher BMI in children drinking >12 ounces daily of SSBs compared to non-drinkers.
Keller et al. [53]	Am. Diet. Assoc. 2009	Cross-sectional	126 children, 3–7 years old	Any type	No difference in BMI according to SSB intake.
Dubois et al. [54]	J. Am. Diet. Assoc. 2007	Longitudinal	1944 children, born in 1998	Carbonated drinks and fruit flavored drinks (excluding 100% fruit juice)	Children who drank SSBs between the ages of 2.5–4.5 years had an OR of 2.4 for being overweight at age 4.5 years compared to non-drinkers.
Te Morenga et al. [55]	BMJ 2012	Meta-analysis	22 randomized controlled trials and cohort studies among children and adults	Any type (including fruit juices)	OR of 1.55 for higher levels of BMI among SSB drinkers over time.
Pan et al. [56]	Pediatrics 2014	Longitudinal	1189 children involved in the Infant Feeding Practices Study II and followed up at 6 years	Juice drinks, soft drinks, soda, sweet tea, Kool-Aid etc.	Odds for obesity detected were 71% higher for any SSB intake and 92% higher for SSB introduction before age 6 months.
Zheng et al. [57]	Eur. J. Clin. Nutr. 2014	Longitudinal	283 Danish children aged 9 years followed up at 6 years and 12 years	Soft drinks, fruit drinks and cordials sweetened with caloric sweeteners (excluding 100% fruit juice, flavored milk, coffee and tea)	Subjects who consumed more than 1 serving of an SSB per day at age 15 years had higher BMI and waist circumference than non-drinkers in the subsequent 6 years. Moreover, children who increased SSB intake from age 9 to 15 years had larger increases in BMI and waist circumference from 15–21 years compared to those with no increase in SSB intake.
Laurson et al. [58]	Acta Paediatr. 2008	Longitudinal	268 children (age at entry 10 years) studied over an 18-month period	Soft drinks	No association between SSB consumption and weight gain.
Forshee et al. [59]	Am. J. Clin. Nutr. 2008	Meta-analysis	12 longitudinal studies among children ages 2–19 years old	Any type	No association between SSB consumption and weight gain.
Ebbeling et al. [60]	N. Engl. J. Med. 2012	Interventional randomized controlled trial	224 adolescents followed up at 1 year	Any type (including 100% fruit juices)	Adolescents who consumed non-caloric beverages gained less weight (−1.9 kg, *p* = 0.04) and had lower BMI (−0.57, *p* = 0.045) compared to those drinking conventional SSBs.
De Ruyter et al. [61]	N. Engl. J. Med. 2012	Interventional randomized controlled trial	641 children, 4–11 years old	Any type	Children randomized to have non-caloric drinks (versus similar SSBs in a blinded fashion) by 18 months gained less weight, had lower BMI z-scores, smaller waist circumferences and lower fat percentages.
Malik et al. [62]	Am. J. Cardiol. 2014	Meta-analysis	12 studies with 409,707 participants (children >12 years and adults)	Any beverage with a caloric sweetener added	Subjects in the highest quantile of SSB intake (1–2 servings/day) had a 26% greater risk of developing T2DM with respect to individuals in the lowest quantile (none or <1 serving/month).
Farhangi et al. [63]	J. Transl. Med. 2020	Systematic review and meta-analysis	14 studies with 93,873 participants (children and adolescents <19 years)	Any type	High SSB consumption was associated with a 1.67 mmHg increase in systolic BP in children and adolescents but not with a significant rise in diastolic BP. High SSB consumers were moreover 1.36 times more likely to develop HTN compared with low SSB consumers.
Pollock et al. [64]	J. Nutr. 2012	Cross-sectional	559 US adolescents, 14–18 years old	Any type (including 100% fruit juices)	Higher total fructose consumption was positively associated with risk for cardiovascular disease and T2DM.
Welsh et al. [65]	Circulation 2011	Cross-sectional	2157 US adolescents	Any type	Positive association between consumption of added sugars with multiple cardiovascular risk markers (high LDL and TG and low HDL levels, (*p* trend = 0.001–0.01).
Zhu et al. [66]	Pediatr. Obes. 2020	Cross-sectional	3958 children and adolescents, 6–17 years old	Any type (excluding fruit juices)	SSB intake was positively correlated with serum total cholesterol and LDL and participants in the highest intake category of SSB consumption had higher total cholesterol and higher LDL levels than the non-consumption group. Moreover, a positive association between consumption of added sugars and BMI (*p* = 0.04) was shown.
Mirmiran et al. [67]	Nutr. Metab. 2015	Longitudinal study	424 children and adolescents, 6–18 years old	Sweetened carbonated soft drinks and fruit juice drinks	Compared to the first quartile of SSB intake, the OR of incident MetS in the highest quartile was 3.20; the OR for abdominal obesity was 2.49 and 2.79 for HTN.
Li et al. [68]	Public Health Nutr. 2020	Cross-sectional	7143 children and adolescents, 7–18 years old	Energy drinks, sports drinks, soda drinks, fruit drinks with added sugar, sweetened tea and coffee drinks	Subjects with high SSB intake were at higher risk of MetS (OR = 1.60) and abdominal obesity (OR = 1.55) compared with their participants with no SSB intake.

## Data Availability

Not applicable.
